# Supplementary material for: Estimating glioblastoma migration-to-proliferation ratio from single time-point MRI data via radial density distribution
Source: bioRxiv. 2026 Oct 30:2026.10.26.761772. Preprint. [Version 1] doi: 10.64898/2026.10.26.761772 (PMC13733866; doi:10.64898/2026.10.26.761772)
Supplement: Supplementary file 1 [file NIHPP2026.10.26.761772V1-supplement-1.pdf]

bioRxiv preprint doi: https://doi.org/10.64898/2026.10.26.761772; this version posted October 31, 2026. The copyright holder for this preprint (which was not certified by peer review) is the author/funder, who has granted bioRxiv a license to display the preprint in perpetuity. It is made available under a CC-BY 4.0 International license.

Supplemental

Table S1: BraTS 2017 patient demographic information. All patients underwent surgery.

**Characteristics**	**Value**
Male (# patients)	63
female (# patients)	38

bioRxiv preprint doi: https://doi.org/10.64898/2026.10.26.761772; this version posted October 31, 2026. The copyright holder for this preprint (which was not certified by peer review) is the author/funder, who has granted bioRxiv a license to display the preprint in perpetuity. It is made available under a CC-BY 4.0 International license.

diagnosis age 0-18 (# patients)	1
diagnosis age 19-44 (# patients)	14
diagnosis age 44-64 (# patients)	44
diagnosis age 64+ (# patients)	30
White race (# patients)	86
Black or African American race (# patients)	7
Asian race (# patients)	3
Hispanic or Latino race (# patients)	1
Median overall survival (months)	12.61
Radiotherapy (# patients)	99
Pharmaceutical Therapy (# patients)	72
Chemotherapy (# patients)	71
Targeted Therapy (# patients)	19
Immunotherapy (# patients)	6
Hormone Therapy (# patients)	4
Radiotherapy + Chemotherapy + Pharmaceutical Therapy (# patients)	34
Radiotherapy + Pharmaceutical Therapy (# patients)	20
Radiotherapy + Chemotherapy (# patients)	16

bioRxiv preprint doi: https://doi.org/10.64898/2026.10.26.761772; this version posted October 31, 2026. The copyright holder for this preprint (which was not certified by peer review) is the author/funder, who has granted bioRxiv a license to display the preprint in perpetuity. It is made available under a CC-BY 4.0 International license.

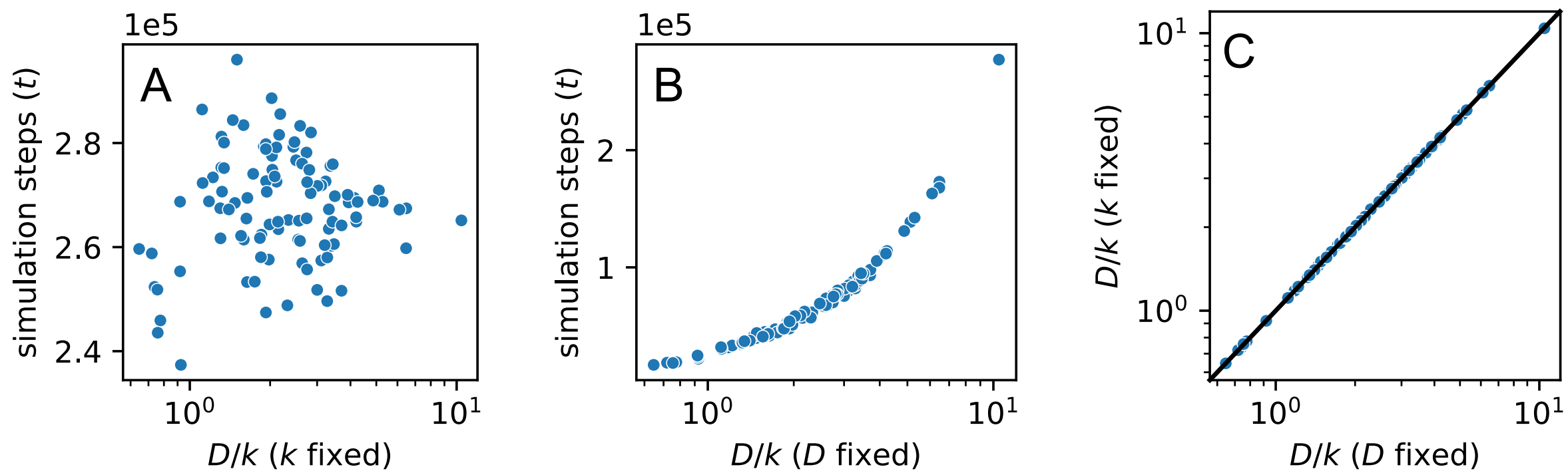

Figure S1: A, B *D*/*k* ratios and optimal numbers of simulation steps calculated for the 102 patients from the BraTS 2017 dataset, with *k* fixed (panel A) and *D* fixed (panel B). **C** *D*/*k* ratios with *k* fixed plotted against *D*/*k* ratios with *D* fixed. CCC = 1.000

Starting the synthetic tumor near the domain boundary distorts the inferred D/k

We also determined the impact of initiating the synthetic tumor near the edge of the computational domain on the ability to accurately estimate *D*/*k* (Figure S2A). For this test, we assessed two different methods for finding the tumor initial position: the center of mass of the enhancing region (Figure S2B) and the intersection point of the two longest perpendicular diameters within the enhancing region (Figure S2C). The center of mass method showed reduced correlation with the synthetic *D*/*k* values when the tumor initial position was within 10 mm of the boundary compared to 20 mm (PCC = 0.927 versus 0.995), and the longest diameters method maintained high correlation regardless of boundary proximity (PCC = 0.997 versus 0.999).

When applied to the patient data, however, we observed that the longest diameters method tended to generate larger *D*/*k* values relative to the center of mass method. This is the opposite of the correction that we observed with the synthetic data (Figure S2D, E). This discrepancy is likely due to the longest perpendicular diameters method selecting tumor initial positions that are farther from the center of the enhancing region in asymmetrical tumors (Figure S2F). We thus chose the center of mass method because of its simplicity and consistency when applied to the patient data.

bioRxiv preprint doi: https://doi.org/10.64898/2026.10.26.761772; this version posted October 31, 2026. The copyright holder for this preprint (which was not certified by peer review) is the author/funder, who has granted bioRxiv a license to display the preprint in perpetuity. It is made available under a CC-BY 4.0 International license.

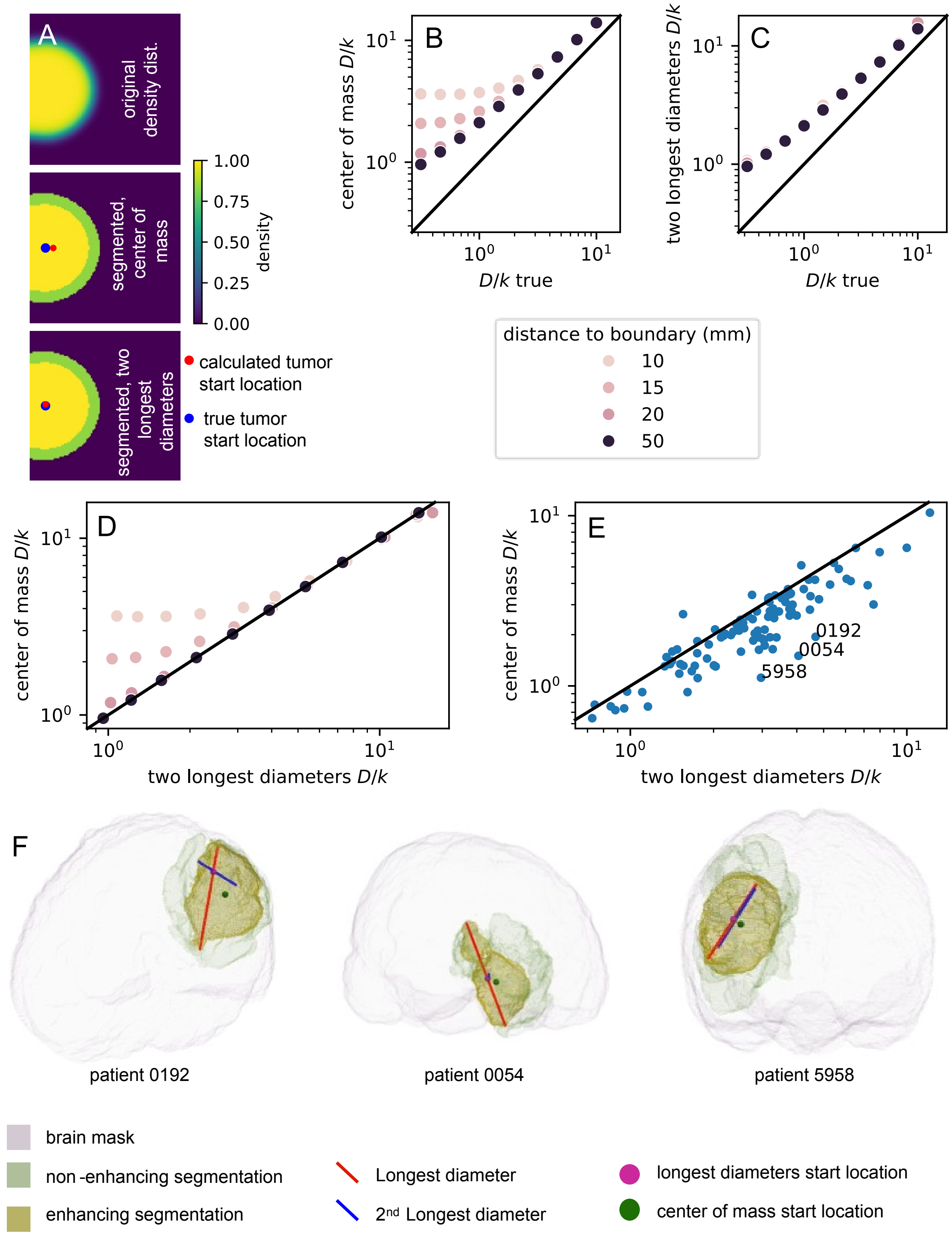

Figure S2: Boundary proximity effects and tumor initial position estimation methods **A**

bioRxiv preprint doi: https://doi.org/10.64898/2026.10.26.761772; this version posted October 31, 2026. The copyright holder for this preprint (which was not certified by peer review) is the author/funder, who has granted bioRxiv a license to display the preprint in perpetuity. It is made available under a CC-BY 4.0 International license.

Top: 2D slice of 3D model run where tumor initial position is near the edge of the domain, middle and bottom: segmented tumor with initial position calculated via center of mass method and longest diameters method. **B, C** *D*/*k* calculated via center of mass method and longest diameters method versus synthetic data *D*/*k* (D/k_{true}) for different distances of the tumor initial position from the boundary. **D, E** *D*/*k* calculated from the two longest diameters method versus the center of mass method for the **D** Synthetic data and **E** 102 patients from the BraTS 2017 dataset. **F** Three patients with particularly large discrepancies in calculated *D*/*k* are plotted in 3D to demonstrate the difference in tumor initial position between the longest diameters method and the center of mass method.

bioRxiv preprint doi: https://doi.org/10.64898/2026.10.26.761772; this version posted October 31, 2026. The copyright holder for this preprint (which was not certified by peer review) is the author/funder, who has granted bioRxiv a license to display the preprint in perpetuity. It is made available under a CC-BY 4.0 International license.

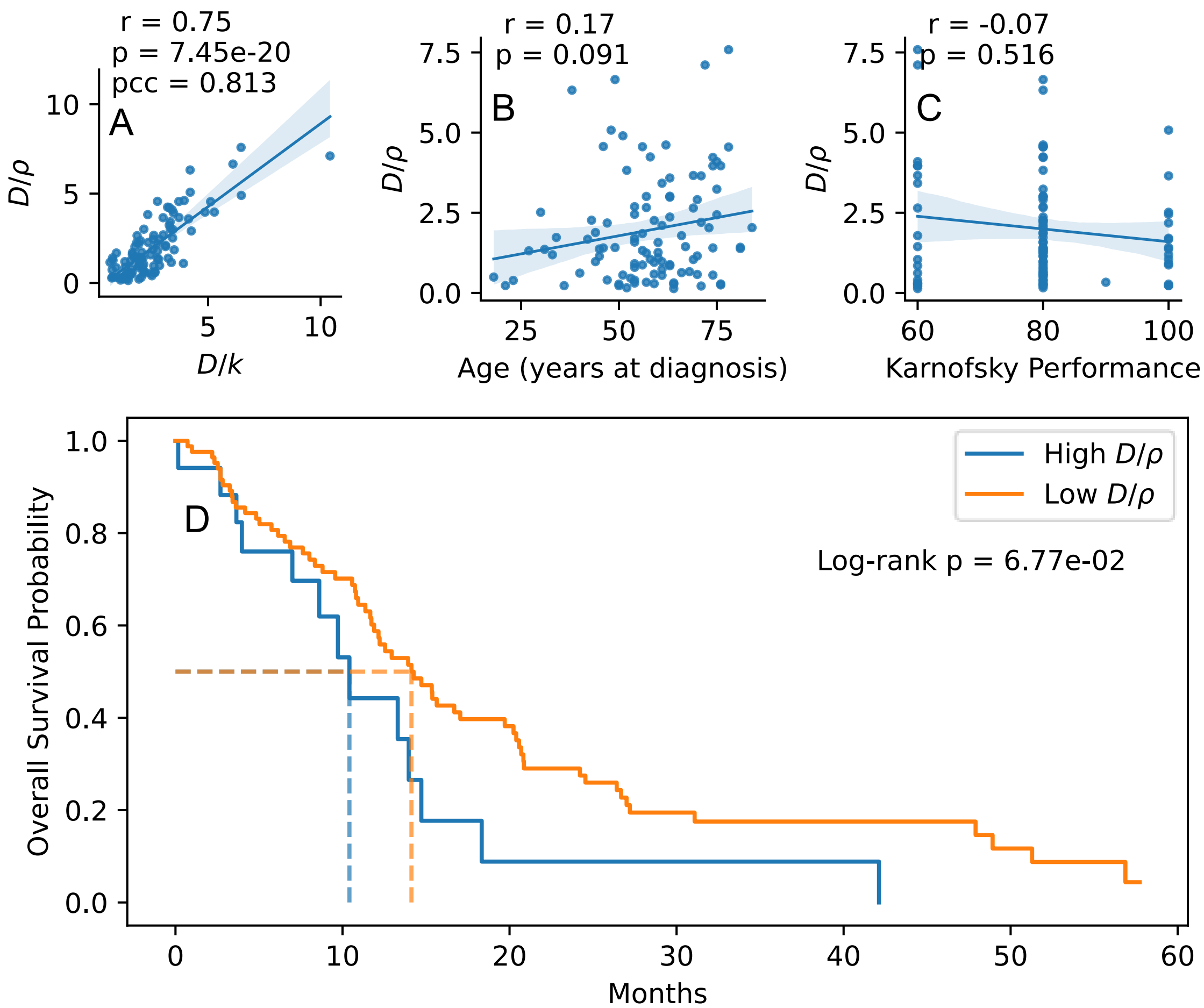

	0	10	20	30	40	50	60
**High D/ρ**							
At risk	17	6	1	1	1	0	0
Censored	0	4	4	4	4	4	4
Events	0	7	12	12	12	13	13
**Low D/ρ**							
At risk	84	50	25	10	8	4	0
Censored	0	10	13	16	17	19	21
Events	0	24	46	58	59	61	63

Figure S3: Comparison of D/k with the Swanson D/ρ method and clinical outcomes. **A** Correlation between D/k and Swanson D/ρ[11] method applied to BraTS patient data. D/ρ found using Equation (1) from Tropeano et al.[12]. **B, C** Correlation of patient age versus D/ρ and Karnofsky Performance Score versus D/ρ. Spearman correlation was used to calculate r and p values. **D** Optimal cut point between 10th and 90th percentiles of the patient data ordered by D/ρ was determined via log-rank test to separate patients into Low D/ρ and High D/ρ groups. Kaplan-Meier curves of optimally cut groups are shown with associated log-rank p-value.

bioRxiv preprint doi: https://doi.org/10.64898/2026.10.26.761772; this version posted October 31, 2026. The copyright holder for this preprint (which was not certified by peer review) is the author/funder, who has granted bioRxiv a license to display the preprint in perpetuity. It is made available under a CC-BY 4.0 International license.

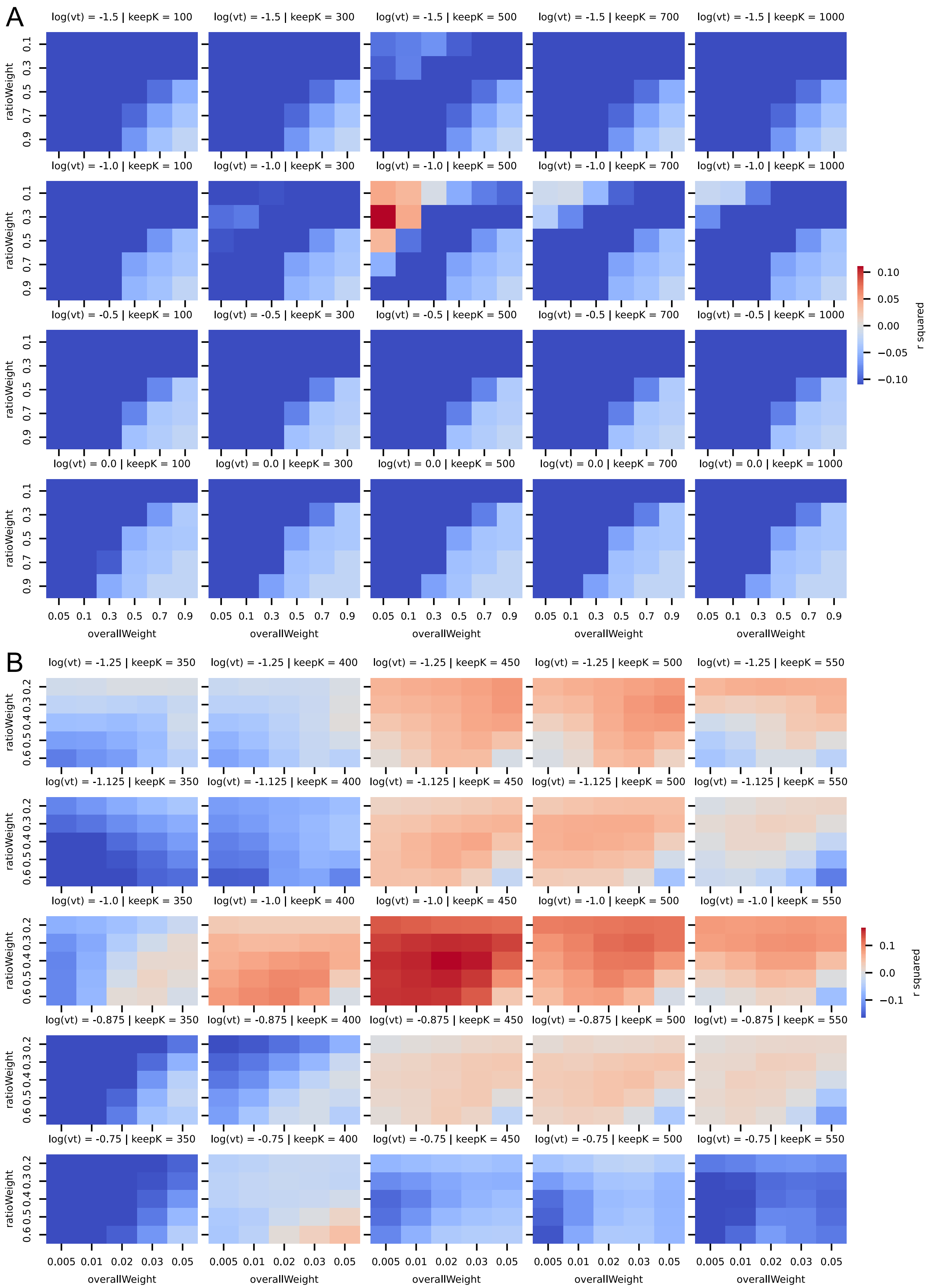

bioRxiv preprint doi: https://doi.org/10.64898/2026.10.26.761772; this version posted October 31, 2026. The copyright holder for this preprint (which was not certified by peer review) is the author/funder, who has granted bioRxiv a license to display the preprint in perpetuity. It is made available under a CC-BY 4.0 International license.

Figure S4: A, B Elastic Net Regression: hyper parameter tuning performance . Within heatmaps: rows indicate weighting of ratio between ridge regression and LASSO regression, columns indicate weighting of combination of ridge and LASSO regression penalty versus ordinary least-squares regression. Across heatmaps: rows indicate gene microarray variance threshold as part of pre-processing, columns indicate the number of genes most correlated with *D*/*k* that are subsequently fed into Elastic Net.

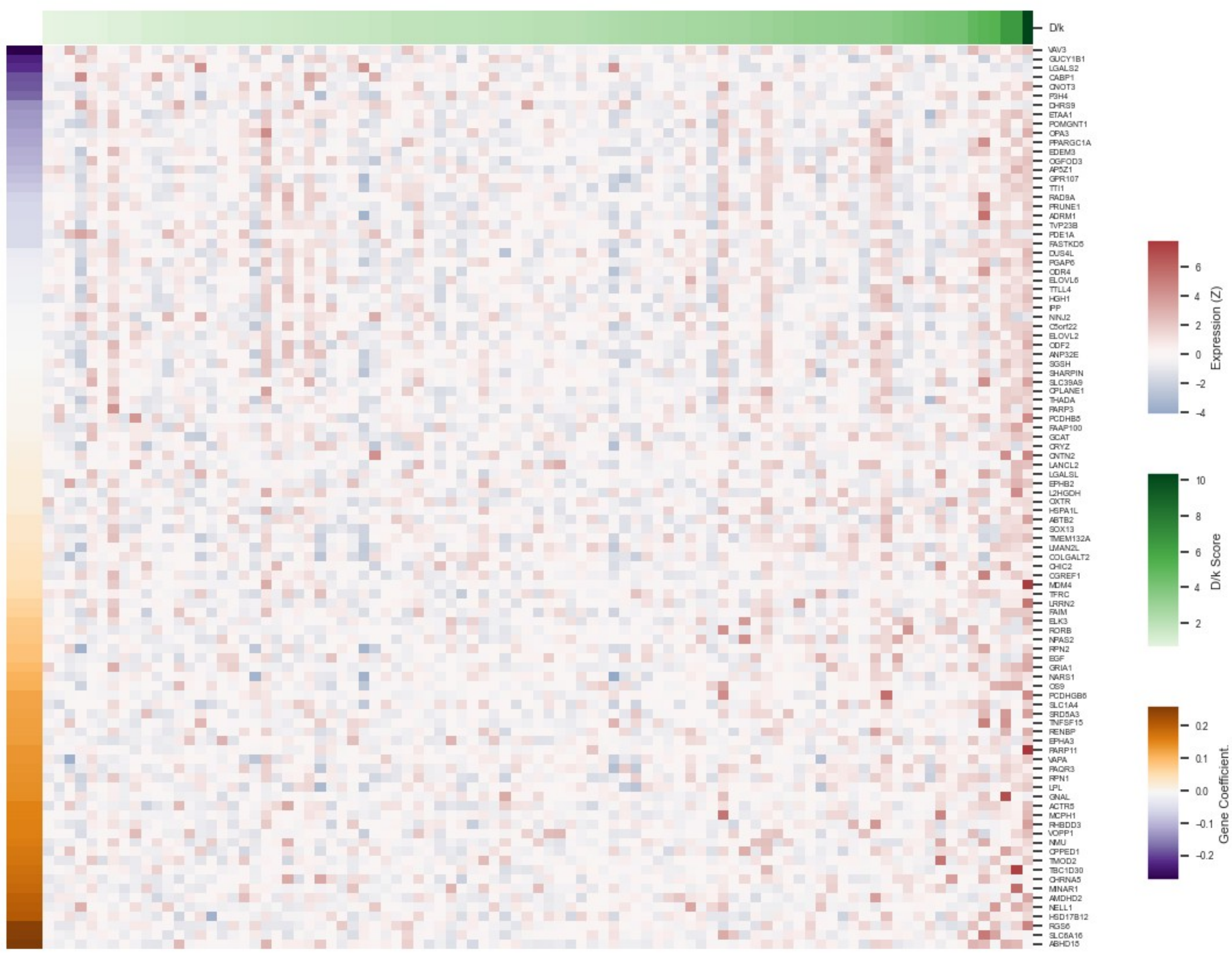

Figure S5: Microarray expression of genes chosen by Elastic Net regression model trained on the full patient dataset. The top annotation bar indicates the estimated *D*/*k* for each patient. Left annotation bar indicates the linear model coefficients.

bioRxiv preprint doi: https://doi.org/10.64898/2026.10.26.761772; this version posted October 31, 2026. The copyright holder for this preprint (which was not certified by peer review) is the author/funder, who has granted bioRxiv a license to display the preprint in perpetuity. It is made available under a CC-BY 4.0 International license.

Gene set analysis results

We performed overrepresentation and GSVA[74] analysis for Reactome pathways and the most positively correlated Reactome pathways with *D*/*k* are listed in Table S3. Many Reactome pathways were significantly correlated with before multiple test correction, they were not significant after.

In overrepresentation analysis, the closest was to the UV_RESPONSE_UP gene set and the MTORC1_SIGNALING gene set. Upregulation of UV response genes could be linked to decreased drug response in elevated- *D*/*k* patients, as the genes in this pathway are associated with DNA repair, which could also dampen response to chemotherapy and radiotherapy. The mTOR signaling reflects multiple cellular processes including proliferation and migration, suggesting that elevated mTOR activity corresponds to more advanced disease.

Table S2: Results from ORA done on genes chosen by Elastic Net regression run on all patients (msigdb database).

Term	Overlap	P-value	Adjusted P-value	Odds Ratio	Combined Score	Genes
HALLMARK_UV_RESPONSE_UP	3/148	0.0222	0.3577	6.1314	23.3517	RPN1;CHRNA5;TFRC
HALLMARK_MTORC1_SIGNALING	3/186	0.0397	0.3577	4.8461	15.63	SLC1A4;RPN1;TFRC
HALLMARK_ANGIOGENESIS	1/36	0.1367	0.5342	10.4218	20.7418	LPL
HALLMARK_ESTROGEN_RESPONSE_LATE	2/189	0.1792	0.5342	3.3152	5.6998	SLC1A4;NMU

bioRxiv preprint doi: https://doi.org/10.64898/2026.10.26.761772; this version posted October 31, 2026. The copyright holder for this preprint (which was not certified by peer review) is the author/funder, who has granted bioRxiv a license to display the preprint in perpetuity. It is made available under a CC-BY 4.0 International license.

Table S3: Reactome pathways whose GSVA scores are most correlated with the estimated *D*/k *.

**Pathway**	**Spearman_ Rho**	**P_Value**
HS-GAG Degradation	0.3178	0.0021
Diseases of Carbohydrate Metabolism	0.3011	0.0037
Diseases of Metabolism	0.2982	0.0041
Diseases of Glycosylation	0.2743	0.0085
Defective EXT2 Causes Exostoses 2	0.2726	0.0089
Defective EXT1 Causes Exostoses 1, TRPS2 and CHDS	0.2726	0.0089
HS-GAG Biosynthesis	0.2606	0.0126
Mucopolysaccharidoses	0.2552	0.0146
Keratan Sulfate Degradation	0.2515	0.0162
Heparan Sulfate Heparin (HS-GAG) Metabolism	0.2493	0.0172

*No significant results after FDR correction.
